# Feasibility of noninvasive 3 T MRI-guided myocardial ablation with high intensity focused ultrasound

**DOI:** 10.1186/1532-429X-11-S1-O86

**Published:** 2009-01-28

**Authors:** Aravind Swaminathan, Viola Rieke, Randy L King, John Pauly, Kim Butts-Pauly, Michael McConnell

**Affiliations:** 1 Stanford Hospital &Clinics, Stanford, CA USA; 2 Stanford University Department of Radiology, Stanford, CA USA; 3 Stanford University Department of Electrical Engineering, Stanford, CA USA

**Keywords:** Hypertrophic Cardiomyopathy, Focus Ultrasound, Acoustic Power, Ablation Lesion, Cardiac Gating

## Objective

This study sought to determine the feasibility of noninvasive myocardial HIFU ablation using real-time MRI guidance and thermometry.

## Background

Invasive catheter-based myocardial ablation has become an important treatment of hypertrophic cardiomyopathy (HCM) and cardiac arrhythmias, but has known complications as well as the inability to actively visualize and control the extent of ablated tissue. High-intensity focused ultrasound (HIFU) can noninvasively create focal ablation lesions and has been developed for multiple non-cardiac clinical applications. MRI, in addition to imaging of myocardial pathology, can provide image guidance of HIFU targeting and then perform real-time monitoring of myocardial temperature during ablation. This study included preliminary feasibility work on ex-vivo MRI-guided myocardial HIFU with cardiac gating.

## Methods

For ex vivo ablation, an existing MRI-guided HIFU ablation system (Insightec Ltd., Tirat Carmel, Israel) was used on a 3 T MRI scanner (GE Healthcare, Milwaukee, WI). Ex-vivo porcine hearts (N = 7) were immersed in water and degassed. MR scout imaging was performed to identify and guide the myocardial treatment areas to the septum. Multiple HIFU ablations lesions were formed using acoustic powers between 60–90 Watts and sonication duration of 20 s at a HIFU frequency of 1.1 MHz. MR thermometry was performed during lesion formation to verify correct ablation location and achievement of thermal ablation threshold (>55°C). T2-weighted imaging was used to image lesions post-ablation. Lesion location and size was confirmed by pathology. Additional experiments were performed to simulate cardiac gating – HIFU pulses (acoustic power 150 W, duration 20 s) were activated once per second (assuming heart rate of 60 bpm) with a range of pulse durations (100 ms–1 s).

## Results

**Figure 1:**
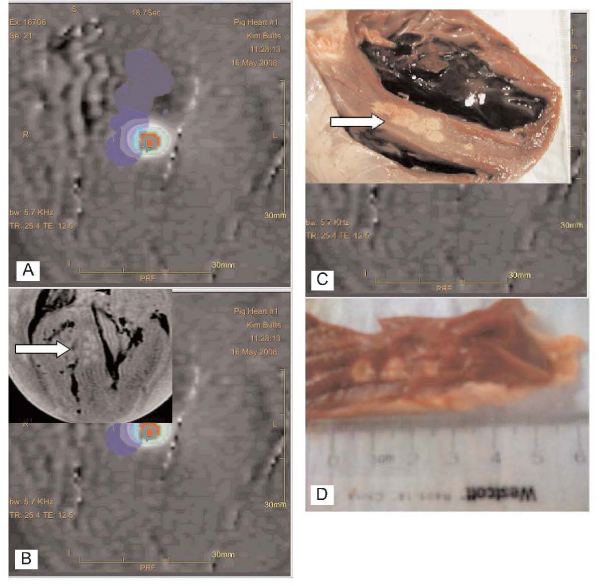
**A) Real-time MR-temperature map during HIFU septal ablation (red zone on image), B) T2 MR image of HIFU lesions, C) Gross pathology of septal lesions from (B) using multiple 20 s pulses, D) Lesions, each 4 mm × 4 mm created with single 20 s HIFU pulses along the LV lateral wall**.

**Figure 2:**
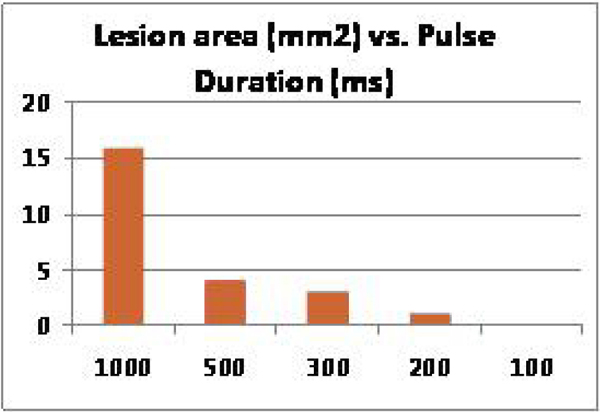
**Lesion size (mm2) as a function of pulse duration. Pulses were cycled on and off with a signal generator at 1 Hz**.

Ablation lesions were formed in the ventricular septum of ex-vivo porcine hearts, with lesion size adjustable depending on the number of sonications used (each 20 s pulse created a 4 mm × 4 mm lesion). (Figure 1a–d). Lesion size decreased at lower pulse duration. Pulse durations as low as 200 ms with interval cooling durations of 800 ms created HIFU lesions as small as 1 mm × 1 mm with no visible lesion at lower duration (Figure 2). This cutoff is compatible with ablation during normal cardiac cycle lengths (600–1000 ms).

## Conclusion

This study shows noninvasive MRI-guided HIFU is feasible on ex-vivo myocardium using a 3 T MRI-guided HIFU system with MR-based temperature monitoring. Furthermore, lesions could be created with HIFU pulses under physiologic cardiac gating intervals. Further work is needed based on these results to allow animal testing and ultimately clinical translation.

